# [(4-Bromo­phen­yl)(2-pyridyl­methyl­idene)amine-κ^2^ *N*,*N*′]bis­(1,1,1,5,5,5-hexa­fluoro­pentane-2,4-dionato-κ^2^ *O*,*O*′)cobalt(II)

**DOI:** 10.1107/S1600536810032757

**Published:** 2010-08-21

**Authors:** Phimphaka Harding, David J. Harding, Nitisastr Soponrat, Harry Adams

**Affiliations:** a Molecular Technology Research Unit, Department of Chemistry, Walailak University, Thasala, Nakhon Si Thammarat 80161, Thailand; b Department of Chemistry, Faculty of Science, Taksin University, Songkhla 90000, Thailand; c Department of Chemistry, Faculty of Science, University of Sheffield, Brook Hill, Sheffield S3 7HF, England

## Abstract

In the title complex, [Co(C_5_HF_6_O_2_)_2_(C_12_H_9_BrN_2_)], the Co^II^ atom exhibits a pseudo-octa­hedral coordination geometry, comprising two *N*-donor atoms from a bidentate chelate (4-bromo­phen­yl)(2-pyridyl­methyl­idene)amine (ppa^Br^) ligand [Co—N = 2.098 (2) and 2.209 (2) Å] and four *O*-donor atoms from two bidentate chelate 1,1,1,5,5,5-hexa­fluoro­pentane-2,4-dionate (hfac) ligands [Co—O range = 2.0452 (19)–2.0796 (19) Å]. The packing of the structure involves weak π–π inter­actions between the pyridyl and benzene rings of neighbouring ppa^Br^ ligands [centroid–centroid distance = 3.928 (2) Å] and inter­actions between the Br atom on the ppa^Br^ ligand and the hfac ligand [Br⋯C = 3.531 (2) Å].

## Related literature

For a review of halogen bonding, see: Corradi *et al.* (2000 ▶); Walsh *et al.* (2001 ▶); Liantonio *et al.* (2003 ▶). For an introduction to crystal engineering, see: Braga *et al.* (2002 ▶). For related structures, see: Harding, Harding, Sophonrat & Adams (2010 ▶); Harding, Harding, Tinpun *et al.* (2010 ▶); Aäkeroy *et al.* (2004 ▶, 2007 ▶). For a description of the Cambridge Structural database, see: Allen *et al.* (2002 ▶).
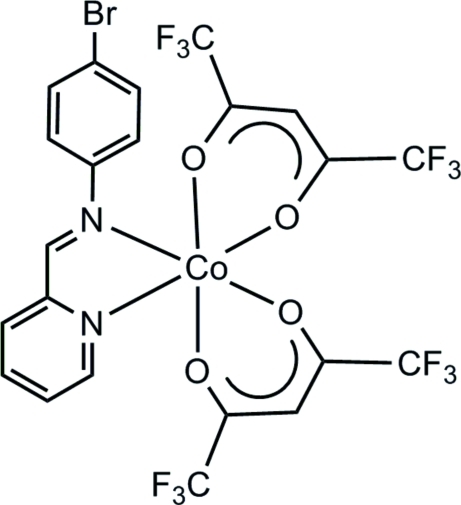


## Experimental

### 

#### Crystal data

[Co(C_5_HF_6_O_2_)_2_(C_12_H_9_BrN_2_)]
*M* *_r_* = 734.17
Triclinic, 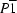

*a* = 8.3568 (2) Å
*b* = 10.9420 (2) Å
*c* = 14.8151 (3) Å
α = 74.042 (1)°
β = 86.510 (1)°
γ = 77.080 (1)°
*V* = 1269.51 (5) Å^3^
*Z* = 2
Mo *K*α radiation
μ = 2.37 mm^−1^
*T* = 150 K
0.60 × 0.30 × 0.03 mm

#### Data collection

Bruker SMART CCD area-detector diffractometer
Absorption correction: multi-scan (*SADABS*; Bruker, 1997 ▶) *T*_min_ = 0.330, *T*_max_ = 0.932
21525 measured reflections
5176 independent reflections
4508 reflections with *I* > 2σ(*I*)
*R*_int_ = 0.021

#### Refinement

*R*[*F* ^2^ > 2σ(*F* ^2^)] = 0.035
*wR*(*F* ^2^) = 0.090
*S* = 1.08
5176 reflections
379 parameters
H-atom parameters constrained
Δρ_max_ = 1.23 e Å^−3^
Δρ_min_ = −0.91 e Å^−3^

### 

Data collection: *SMART* (Bruker, 1997 ▶); cell refinement: *SAINT* (Bruker, 1997 ▶); data reduction: *SAINT*; program(s) used to solve structure: *SHELXS97* (Sheldrick, 2008 ▶); program(s) used to refine structure: *SHELXL97* (Sheldrick, 2008 ▶); molecular graphics: *SHELXTL* (Sheldrick, 2008 ▶); software used to prepare material for publication: *SHELXTL*.

## Supplementary Material

Crystal structure: contains datablocks I, global. DOI: 10.1107/S1600536810032757/zs2056sup1.cif

Structure factors: contains datablocks I. DOI: 10.1107/S1600536810032757/zs2056Isup2.hkl

Additional supplementary materials:  crystallographic information; 3D view; checkCIF report

## Figures and Tables

**Fig. 1.**
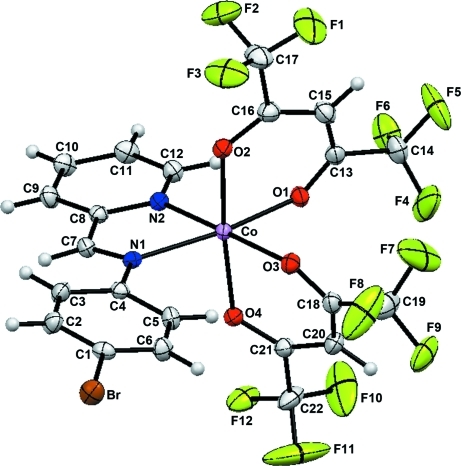
The molecular structure of (I) showing the atom-labelling scheme. Displacement ellipsoids are drawn at the 50% probability level.

**Fig. 2.**
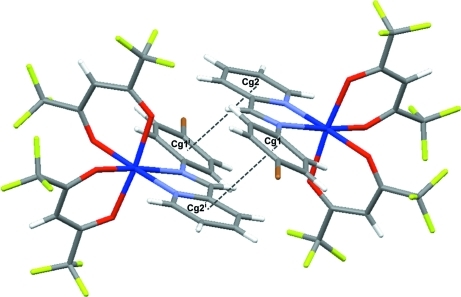
The molecular packing in (I) showing the π–π interactions between the phenyl and pyridyl rings of the ppaBr ligand. Only selected atoms are labelled for clarity. [Symmetry code: (i) -x + 2, -y, -z + 2].

**Fig. 3.**
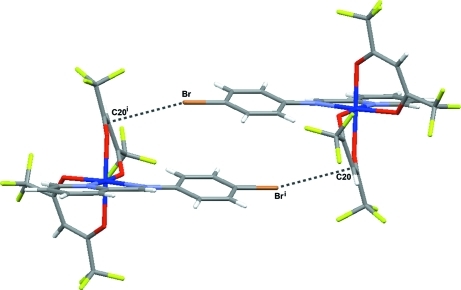
The molecular packing in (I) showing the Br···β-diketonate interactions of the discrete dimers. Only selected atoms are labelled for clarity. [Symmetry code: (i) -x + 1, -y + 1, -z + 2].

## supplementary crystallographic information

### Comment

The construction of supramolecular networks with designed architectures still remains the goal of crystal engineering and represents a significant challenge (Braga *et al.*, 2002). Although complementary hydrogen-bonding ligands have been successfully used (Aäkeroy *et al.*, 2004) in the construction of a number of networks, halogen-bonding (Walsh *et al.*, 2001; Liantonio *et al.*, 2003) and halogen···halogen interactions remain less well represented despite the fact that these interactions can be as strong as hydrogen-bonding interactions (Corradi *et al.*, 2000). In this paper we report the synthesis and structure of [Co(hfac)_2_(ppa^Br^)] [hfac = 1,1,1,5,5,5-hexafluoropentane-2,4-dionato; ppa^Br^ = (4-bromo-phenyl)pyridin-2-ylmethyleneamine].

The reaction of [Co(hfac)_2_(H_2_O)_2_] with ppa^Br^ in CH_2_Cl_2_ yields [Co(hfac)_2_(ppa^Br^)] (I) (Fig. 1) which crystallizes from CH_2_Cl_2_/hexane. In (I) the cobalt metal centre is six-coordinate with a distorted octahedral geometry, the hfac ligands adopting a *cis* arrangement enforced by the chelating ppa^Br^ ligand. The CF_3_ groups of the hfac ligand in some cases exhibit large thermal ellipsoids due to thermal motion of these groups. The Co—N and Co—O bond lengths are comparable with related cobalt hfac and diimine complexes reported in the CSD (Allen, 2002) (mean Co—O distance = 2.01 Å, Co—N distance = 2.11 Å). The β-diketonate ligands exhibit a *bent* coordination mode in which the angles between the planes defined by the Co and oxygen atoms and the carbon and oxygen atoms of the β-diketonate ligand are 18.9° and 24.7°. In contrast, in *trans*-[*M*(hfac)_2_(py-CH=CH—C_6_F_4_Br)_2_] (*M* = Co, Cu) the β-diketonate ligands exhibit a *planar* coordination mode (Aäkeroy *et al.*, 2007). In addition, the phenyl ring is twisted with respect to the pyridylimine unit by 17.6° and is similar to the angle observed in [Ni(dbm)_2_(ppa*^X^*)] [*X* = Me, 22.9°; Cl, 24.0° (Harding, Harding, Tinpun *et al.*, 2010)].

The packing in the structure of (I) involves a weak π–π interaction between the pyridyl and phenyl rings of neighbouring ppa^Br^ ligands as shown in Fig. 2 (*Cg*1···*Cg*2 = 3.928 (2) Å where *Cg*1 and *Cg*2 are the centroids of the rings C1—C6 and C8—C12—N2). A further weak interaction occurs between the Br atom on the ppa^Br^ ligand and the β-diketonate ligand creating discrete dimers within the structure [Br···C20, 3.531 (2) Å, see Fig. 3]. These dimers are then connected *via* the π–π interaction mentioned above resulting in one-dimensional chains. A similar interaction is also observed in the structure of *trans*-[*M*(hfac)_2_(py-CH=CH—C_6_F_4_Br)_2_] (Aäkeroy *et al.*, 2007). Interestingly, the corresponding Ni analogue, [Ni(hfac)_2_(ppa^Br^)] has a completely different set of interactions with Br···CH interactions clearly evident (Harding, Harding, Sophonrat & Adams, 2010), once again highlighting the difficulties involved in attempting to use specific interactions in the design of supramolecular networks.

### Experimental

To an orange red solution of [Co(hfac)_2_(H_2_O)_2_] (0.127 g, 0.25 mmol) in CH_2_Cl_2_ (5 cm^3^) was added a solution of ppa^Br^ (0.065 g, 0.25 mmol) in CH_2_Cl_2_ (3 cm^3^). The deep orange solution was stirred for 1 h and then concentrated *in vacuo*. *n*-Hexane (15 cm^3^) was added to precipitate an orange solid which was washed with *n*-hexane (2 *x* 5 cm^3^) and dried *in vacuo*: yield 0.142 g (77%). IR in KBr disc ν_C=O_ 1647 cm^-1^. UV-Vis (in CH_2_Cl_2_, log ε mol.dm^-3^cm^-1^) 243 (4.24), 309 (4.42). C_22_H_11_O_4_N_2_F_12_BrCo; calc. C 36.0, H 1.5, N 3.8%; found C 36.5, H 1.5, N 3.8%.

### Refinement

Hydrogen atoms were placed geometrically and refined using a riding model with C–H = 0.95 Å and *U*_iso_ constrained to be 1.2 times *U*_eq_ of the carrier atom.

### Crystal data

**Table:**

[Co(C_5_HF_6_O_2_)_2_(C_12_H_9_BrN_2_)]	*Z* = 2
*M**_r_* = 734.17	*F*(000) = 718
Triclinic, *P*1	*D*_x_ = 1.921 Mg m^−^^3^
Hall symbol: -P 1	Mo *K*α radiation, λ = 0.71073 Å
*a* = 8.3568 (2) Å	Cell parameters from 9953 reflections
*b* = 10.9420 (2) Å	θ = 2.9–32.8°
*c* = 14.8151 (3) Å	µ = 2.37 mm^−^^1^
α = 74.042 (1)°	*T* = 150 K
β = 86.510 (1)°	Plate, orange
γ = 77.080 (1)°	0.60 × 0.30 × 0.03 mm
*V* = 1269.51 (5) Å^3^	

### Data collection

**Table:**

Bruker SMART CCD area-detector diffractometer	5176 independent reflections
Radiation source: fine-focus sealed tube	4508 reflections with *I* > 2σ(*I*)
graphite	*R*_int_ = 0.021
φ and ω scans	θ_max_ = 26.4°, θ_min_ = 2.0°
Absorption correction: multi-scan (*SADABS*; Bruker, 1997)	*h* = −10→10
*T*_min_ = 0.330, *T*_max_ = 0.932	*k* = −13→13
21525 measured reflections	*l* = −18→18

### Refinement

**Table:**

Refinement on *F*^2^	Primary atom site location: structure-invariant direct methods
Least-squares matrix: full	Secondary atom site location: difference Fourier map
*R*[*F*^2^ > 2σ(*F*^2^)] = 0.035	Hydrogen site location: inferred from neighbouring sites
*wR*(*F*^2^) = 0.090	H-atom parameters constrained
*S* = 1.08	*w* = 1/[σ^2^(*F*_o_^2^) + (0.0399*P*)^2^ + 2.417*P*] where *P* = (*F*_o_^2^ + 2*F*_c_^2^)/3
5176 reflections	(Δ/σ)_max_ = 0.001
379 parameters	Δρ_max_ = 1.23 e Å^−^^3^
0 restraints	Δρ_min_ = −0.91 e Å^−^^3^

### Special details

**Table:**

Geometry. All e.s.d.'s (except the e.s.d. in the dihedral angle between two l.s. planes) are estimated using the full covariance matrix. The cell e.s.d.'s are taken into account individually in the estimation of e.s.d.'s in distances, angles and torsion angles; correlations between e.s.d.'s in cell parameters are only used when they are defined by crystal symmetry. An approximate (isotropic) treatment of cell e.s.d.'s is used for estimating e.s.d.'s involving l.s. planes.
Refinement. Refinement of *F*^2^ against ALL reflections. The weighted *R*-factor *wR* and goodness of fit *S* are based on *F*^2^, conventional *R*-factors *R* are based on *F*, with *F* set to zero for negative *F*^2^. The threshold expression of *F*^2^ > σ(*F*^2^) is used only for calculating *R*-factors(gt) *etc*. and is not relevant to the choice of reflections for refinement. *R*-factors based on *F*^2^ are statistically about twice as large as those based on *F*, and *R*- factors based on ALL data will be even larger.

### Fractional atomic coordinates and isotropic or equivalent isotropic displacement parameters (Å^2^)

**Table:**

	*x*	*y*	*z*	*U*_iso_*/*U*_eq_	
Br1	0.24966 (4)	0.40512 (3)	1.12788 (2)	0.02627 (10)	
C1	0.4059 (3)	0.3017 (3)	1.0652 (2)	0.0190 (6)	
C2	0.4540 (4)	0.1698 (3)	1.1069 (2)	0.0234 (6)	
H2	0.4096	0.1318	1.1659	0.028*	
C3	0.5671 (4)	0.0937 (3)	1.0621 (2)	0.0220 (6)	
H3	0.6002	0.0028	1.0903	0.026*	
C4	0.6331 (3)	0.1491 (3)	0.97587 (19)	0.0165 (5)	
C5	0.5813 (3)	0.2824 (3)	0.93439 (19)	0.0198 (6)	
H5	0.6237	0.3208	0.8750	0.024*	
C6	0.4680 (4)	0.3591 (3)	0.9796 (2)	0.0218 (6)	
H6	0.4337	0.4500	0.9519	0.026*	
C7	0.8328 (3)	−0.0354 (3)	0.96759 (19)	0.0182 (5)	
H7	0.8186	−0.0694	1.0331	0.022*	
C8	0.9486 (3)	−0.1115 (3)	0.91526 (19)	0.0172 (5)	
C9	1.0341 (4)	−0.2363 (3)	0.9576 (2)	0.0221 (6)	
H9	1.0201	−0.2753	1.0225	0.027*	
C10	1.1404 (4)	−0.3031 (3)	0.9032 (2)	0.0240 (6)	
H10	1.1991	−0.3896	0.9301	0.029*	
C11	1.1601 (4)	−0.2431 (3)	0.8099 (2)	0.0242 (6)	
H11	1.2336	−0.2869	0.7718	0.029*	
C12	1.0707 (4)	−0.1171 (3)	0.7723 (2)	0.0217 (6)	
H12	1.0847	−0.0757	0.7078	0.026*	
C13	0.8228 (4)	0.1729 (3)	0.5708 (2)	0.0215 (6)	
C14	0.9068 (4)	0.2216 (3)	0.4767 (2)	0.0322 (7)	
C15	0.6657 (4)	0.1503 (3)	0.5689 (2)	0.0258 (6)	
H15	0.6125	0.1698	0.5102	0.031*	
C16	0.5849 (4)	0.0998 (3)	0.6508 (2)	0.0246 (6)	
C17	0.4252 (4)	0.0581 (4)	0.6393 (2)	0.0361 (8)	
C18	0.7556 (4)	0.4215 (3)	0.6922 (2)	0.0223 (6)	
C19	0.6383 (5)	0.5432 (3)	0.6348 (3)	0.0414 (9)	
C20	0.9177 (4)	0.4284 (3)	0.7033 (2)	0.0231 (6)	
H20	0.9542	0.5051	0.6707	0.028*	
C21	1.0267 (4)	0.3261 (3)	0.7608 (2)	0.0203 (6)	
C22	1.1961 (4)	0.3521 (3)	0.7732 (3)	0.0330 (8)	
Co1	0.82077 (4)	0.13568 (3)	0.77538 (2)	0.01482 (10)	
F1	0.3604 (3)	0.1057 (3)	0.55597 (18)	0.0696 (8)	
F2	0.4576 (3)	−0.0730 (2)	0.65283 (19)	0.0578 (7)	
F3	0.3172 (3)	0.0779 (3)	0.70502 (19)	0.0578 (7)	
F4	0.9544 (4)	0.3293 (3)	0.47281 (18)	0.0768 (10)	
F5	0.8123 (3)	0.2441 (3)	0.40289 (14)	0.0636 (8)	
F6	1.0401 (3)	0.1358 (2)	0.46507 (15)	0.0508 (6)	
F7	0.5963 (6)	0.5260 (3)	0.5585 (3)	0.130 (2)	
F8	0.5029 (3)	0.5703 (2)	0.6848 (3)	0.0862 (11)	
F9	0.6978 (3)	0.64976 (19)	0.61453 (18)	0.0505 (6)	
F10	1.2729 (3)	0.3815 (4)	0.6912 (2)	0.0974 (13)	
F11	1.1845 (4)	0.4500 (3)	0.8083 (3)	0.1054 (15)	
F12	1.2953 (2)	0.25146 (19)	0.82617 (16)	0.0406 (5)	
N1	0.7503 (3)	0.0775 (2)	0.92465 (16)	0.0159 (5)	
N2	0.9660 (3)	−0.0524 (2)	0.82359 (16)	0.0171 (5)	
O1	0.9099 (2)	0.15687 (19)	0.64017 (13)	0.0195 (4)	
O2	0.6285 (2)	0.07571 (19)	0.73472 (14)	0.0207 (4)	
O3	0.6895 (2)	0.32603 (18)	0.72450 (13)	0.0194 (4)	
O4	1.0045 (2)	0.21558 (18)	0.80721 (13)	0.0192 (4)	

### Atomic displacement parameters (Å^2^)

**Table:**

	*U*^11^	*U*^22^	*U*^33^	*U*^12^	*U*^13^	*U*^23^
Br1	0.02487 (16)	0.02549 (16)	0.02822 (17)	−0.00312 (12)	0.00920 (12)	−0.01065 (12)
C1	0.0152 (13)	0.0234 (14)	0.0211 (14)	−0.0036 (11)	0.0022 (11)	−0.0116 (11)
C2	0.0295 (16)	0.0212 (14)	0.0213 (14)	−0.0111 (12)	0.0086 (12)	−0.0061 (12)
C3	0.0280 (15)	0.0158 (13)	0.0219 (14)	−0.0066 (12)	0.0031 (12)	−0.0036 (11)
C4	0.0159 (13)	0.0189 (13)	0.0166 (13)	−0.0056 (11)	−0.0007 (10)	−0.0061 (10)
C5	0.0201 (14)	0.0212 (14)	0.0152 (13)	−0.0026 (11)	0.0008 (11)	−0.0021 (11)
C6	0.0222 (14)	0.0179 (13)	0.0214 (14)	0.0010 (11)	−0.0018 (11)	−0.0026 (11)
C7	0.0207 (14)	0.0194 (13)	0.0140 (13)	−0.0048 (11)	−0.0011 (11)	−0.0032 (10)
C8	0.0172 (13)	0.0169 (13)	0.0179 (13)	−0.0040 (11)	−0.0035 (10)	−0.0044 (10)
C9	0.0240 (15)	0.0202 (14)	0.0192 (14)	−0.0010 (12)	−0.0058 (11)	−0.0026 (11)
C10	0.0232 (15)	0.0190 (13)	0.0269 (15)	0.0036 (12)	−0.0074 (12)	−0.0063 (12)
C11	0.0207 (14)	0.0244 (14)	0.0270 (15)	0.0004 (12)	−0.0010 (12)	−0.0102 (12)
C12	0.0224 (14)	0.0227 (14)	0.0195 (14)	−0.0036 (12)	−0.0001 (11)	−0.0057 (11)
C13	0.0279 (15)	0.0176 (13)	0.0181 (14)	−0.0028 (12)	0.0005 (12)	−0.0050 (11)
C14	0.0380 (19)	0.0381 (18)	0.0193 (15)	−0.0089 (15)	0.0039 (13)	−0.0058 (13)
C15	0.0278 (16)	0.0287 (15)	0.0206 (15)	−0.0050 (13)	−0.0044 (12)	−0.0060 (12)
C16	0.0245 (15)	0.0239 (14)	0.0269 (16)	−0.0075 (12)	−0.0044 (12)	−0.0065 (12)
C17	0.0330 (18)	0.050 (2)	0.0322 (18)	−0.0197 (16)	−0.0029 (15)	−0.0127 (16)
C18	0.0281 (16)	0.0191 (14)	0.0175 (14)	−0.0024 (12)	−0.0025 (12)	−0.0030 (11)
C19	0.041 (2)	0.0228 (16)	0.054 (2)	−0.0067 (15)	−0.0224 (18)	0.0045 (16)
C20	0.0279 (15)	0.0195 (14)	0.0218 (14)	−0.0095 (12)	0.0018 (12)	−0.0025 (11)
C21	0.0216 (14)	0.0208 (14)	0.0206 (14)	−0.0074 (11)	0.0005 (11)	−0.0069 (11)
C22	0.0295 (17)	0.0220 (15)	0.048 (2)	−0.0120 (14)	−0.0081 (15)	−0.0028 (14)
Co1	0.01588 (19)	0.01408 (18)	0.01382 (18)	−0.00367 (14)	−0.00033 (14)	−0.00219 (14)
F1	0.0544 (16)	0.104 (2)	0.0513 (15)	−0.0445 (16)	−0.0281 (12)	0.0049 (14)
F2	0.0614 (16)	0.0504 (14)	0.0774 (18)	−0.0309 (12)	0.0001 (13)	−0.0281 (13)
F3	0.0297 (11)	0.0890 (19)	0.0739 (17)	−0.0283 (12)	0.0120 (11)	−0.0436 (15)
F4	0.141 (3)	0.0613 (16)	0.0414 (14)	−0.0620 (18)	0.0404 (16)	−0.0119 (12)
F5	0.0408 (13)	0.118 (2)	0.0176 (10)	−0.0071 (14)	−0.0015 (9)	−0.0019 (12)
F6	0.0361 (12)	0.0704 (16)	0.0345 (12)	0.0019 (11)	0.0130 (9)	−0.0088 (11)
F7	0.238 (5)	0.0398 (15)	0.102 (3)	0.010 (2)	−0.137 (3)	−0.0037 (16)
F8	0.0272 (13)	0.0385 (14)	0.159 (3)	0.0060 (11)	0.0007 (16)	0.0188 (17)
F9	0.0464 (13)	0.0239 (10)	0.0674 (16)	−0.0076 (9)	−0.0113 (11)	0.0128 (10)
F10	0.0476 (16)	0.158 (3)	0.0688 (19)	−0.062 (2)	−0.0036 (14)	0.031 (2)
F11	0.0547 (17)	0.0586 (17)	0.230 (4)	0.0108 (14)	−0.065 (2)	−0.088 (2)
F12	0.0260 (10)	0.0309 (10)	0.0644 (14)	−0.0070 (8)	−0.0163 (10)	−0.0077 (10)
N1	0.0160 (11)	0.0154 (11)	0.0174 (11)	−0.0043 (9)	−0.0008 (9)	−0.0055 (9)
N2	0.0179 (11)	0.0164 (11)	0.0174 (11)	−0.0047 (9)	−0.0002 (9)	−0.0040 (9)
O1	0.0210 (10)	0.0214 (10)	0.0155 (9)	−0.0057 (8)	0.0001 (8)	−0.0031 (8)
O2	0.0224 (10)	0.0206 (10)	0.0195 (10)	−0.0090 (8)	−0.0007 (8)	−0.0025 (8)
O3	0.0192 (10)	0.0179 (9)	0.0194 (10)	−0.0035 (8)	−0.0010 (8)	−0.0026 (8)
O4	0.0209 (10)	0.0181 (9)	0.0190 (10)	−0.0064 (8)	−0.0022 (8)	−0.0031 (8)

### Geometric parameters (Å, °)

**Table:**

Br1—C1	1.902 (3)	C14—F6	1.322 (4)
C1—C6	1.378 (4)	C14—F5	1.322 (4)
C1—C2	1.382 (4)	C15—C16	1.391 (4)
C2—C3	1.379 (4)	C15—H15	0.9500
C2—H2	0.9500	C16—O2	1.256 (4)
C3—C4	1.394 (4)	C16—C17	1.537 (4)
C3—H3	0.9500	C17—F1	1.300 (4)
C4—C5	1.397 (4)	C17—F3	1.316 (4)
C4—N1	1.429 (3)	C17—F2	1.358 (4)
C5—C6	1.388 (4)	C18—O3	1.255 (4)
C5—H5	0.9500	C18—C20	1.396 (4)
C6—H6	0.9500	C18—C19	1.534 (4)
C7—N1	1.284 (4)	C19—F7	1.278 (5)
C7—C8	1.461 (4)	C19—F9	1.321 (4)
C7—H7	0.9500	C19—F8	1.336 (5)
C8—N2	1.349 (4)	C20—C21	1.387 (4)
C8—C9	1.384 (4)	C20—H20	0.9500
C9—C10	1.385 (4)	C21—O4	1.264 (3)
C9—H9	0.9500	C21—C22	1.537 (4)
C10—C11	1.375 (4)	C22—F11	1.296 (4)
C10—H10	0.9500	C22—F12	1.308 (4)
C11—C12	1.392 (4)	C22—F10	1.332 (5)
C11—H11	0.9500	Co1—O2	2.0452 (19)
C12—N2	1.335 (4)	Co1—O4	2.0639 (19)
C12—H12	0.9500	Co1—O1	2.0644 (19)
C13—O1	1.246 (3)	Co1—O3	2.0796 (19)
C13—C15	1.392 (4)	Co1—N2	2.098 (2)
C13—C14	1.537 (4)	Co1—N1	2.209 (2)
C14—F4	1.312 (4)		
			
C6—C1—C2	121.5 (3)	F1—C17—F2	104.7 (3)
C6—C1—Br1	119.8 (2)	F3—C17—F2	103.7 (3)
C2—C1—Br1	118.7 (2)	F1—C17—C16	114.6 (3)
C3—C2—C1	119.3 (3)	F3—C17—C16	112.2 (3)
C3—C2—H2	120.4	F2—C17—C16	109.1 (3)
C1—C2—H2	120.4	O3—C18—C20	128.0 (3)
C2—C3—C4	120.5 (3)	O3—C18—C19	113.7 (3)
C2—C3—H3	119.7	C20—C18—C19	118.3 (3)
C4—C3—H3	119.7	F7—C19—F9	108.7 (4)
C3—C4—C5	119.2 (3)	F7—C19—F8	108.4 (4)
C3—C4—N1	124.2 (2)	F9—C19—F8	104.6 (3)
C5—C4—N1	116.6 (2)	F7—C19—C18	111.1 (3)
C6—C5—C4	120.3 (3)	F9—C19—C18	114.0 (3)
C6—C5—H5	119.9	F8—C19—C18	109.6 (3)
C4—C5—H5	119.9	C21—C20—C18	121.5 (3)
C1—C6—C5	119.2 (3)	C21—C20—H20	119.3
C1—C6—H6	120.4	C18—C20—H20	119.3
C5—C6—H6	120.4	O4—C21—C20	129.1 (3)
N1—C7—C8	119.7 (2)	O4—C21—C22	115.2 (3)
N1—C7—H7	120.1	C20—C21—C22	115.7 (3)
C8—C7—H7	120.1	F11—C22—F12	108.3 (3)
N2—C8—C9	122.5 (3)	F11—C22—F10	106.4 (4)
N2—C8—C7	115.7 (2)	F12—C22—F10	105.6 (3)
C9—C8—C7	121.7 (3)	F11—C22—C21	111.6 (3)
C10—C9—C8	118.5 (3)	F12—C22—C21	113.1 (3)
C10—C9—H9	120.7	F10—C22—C21	111.3 (3)
C8—C9—H9	120.7	O2—Co1—O4	173.89 (8)
C11—C10—C9	119.3 (3)	O2—Co1—O1	87.88 (8)
C11—C10—H10	120.3	O4—Co1—O1	89.87 (8)
C9—C10—H10	120.3	O2—Co1—O3	87.74 (8)
C10—C11—C12	119.0 (3)	O4—Co1—O3	86.41 (8)
C10—C11—H11	120.5	O1—Co1—O3	85.05 (8)
C12—C11—H11	120.5	O2—Co1—N2	94.96 (8)
N2—C12—C11	122.3 (3)	O4—Co1—N2	90.82 (8)
N2—C12—H12	118.9	O1—Co1—N2	92.91 (8)
C11—C12—H12	118.9	O3—Co1—N2	176.56 (8)
O1—C13—C15	128.6 (3)	O2—Co1—N1	91.80 (8)
O1—C13—C14	113.3 (3)	O4—Co1—N1	91.41 (8)
C15—C13—C14	118.1 (3)	O1—Co1—N1	169.94 (8)
F4—C14—F6	106.5 (3)	O3—Co1—N1	104.98 (8)
F4—C14—F5	107.8 (3)	N2—Co1—N1	77.10 (9)
F6—C14—F5	106.3 (3)	C7—N1—C4	119.2 (2)
F4—C14—C13	111.0 (3)	C7—N1—Co1	111.94 (18)
F6—C14—C13	111.4 (3)	C4—N1—Co1	128.79 (17)
F5—C14—C13	113.5 (3)	C12—N2—C8	118.4 (2)
C16—C15—C13	121.6 (3)	C12—N2—Co1	126.20 (19)
C16—C15—H15	119.2	C8—N2—Co1	115.41 (18)
C13—C15—H15	119.2	C13—O1—Co1	123.71 (19)
O2—C16—C15	129.1 (3)	C16—O2—Co1	123.95 (19)
O2—C16—C17	114.0 (3)	C18—O3—Co1	123.68 (19)
C15—C16—C17	116.7 (3)	C21—O4—Co1	122.51 (18)
F1—C17—F3	111.6 (3)		
			
C6—C1—C2—C3	0.2 (4)	C5—C4—N1—C7	162.6 (3)
Br1—C1—C2—C3	179.9 (2)	C3—C4—N1—Co1	163.1 (2)
C1—C2—C3—C4	0.4 (4)	C5—C4—N1—Co1	−15.6 (3)
C2—C3—C4—C5	−1.1 (4)	O2—Co1—N1—C7	97.18 (19)
C2—C3—C4—N1	−179.8 (3)	O4—Co1—N1—C7	−88.02 (19)
C3—C4—C5—C6	1.4 (4)	O1—Co1—N1—C7	9.2 (6)
N1—C4—C5—C6	−179.8 (2)	O3—Co1—N1—C7	−174.68 (18)
C2—C1—C6—C5	0.1 (4)	N2—Co1—N1—C7	2.50 (18)
Br1—C1—C6—C5	−179.7 (2)	O2—Co1—N1—C4	−84.5 (2)
C4—C5—C6—C1	−0.9 (4)	O4—Co1—N1—C4	90.3 (2)
N1—C7—C8—N2	3.1 (4)	O1—Co1—N1—C4	−172.5 (4)
N1—C7—C8—C9	−176.7 (3)	O3—Co1—N1—C4	3.6 (2)
N2—C8—C9—C10	−0.5 (4)	N2—Co1—N1—C4	−179.2 (2)
C7—C8—C9—C10	179.2 (3)	C11—C12—N2—C8	0.8 (4)
C8—C9—C10—C11	1.2 (4)	C11—C12—N2—Co1	−178.8 (2)
C9—C10—C11—C12	−0.9 (4)	C9—C8—N2—C12	−0.5 (4)
C10—C11—C12—N2	−0.2 (4)	C7—C8—N2—C12	179.8 (2)
O1—C13—C14—F4	−55.7 (4)	C9—C8—N2—Co1	179.2 (2)
C15—C13—C14—F4	125.5 (3)	C7—C8—N2—Co1	−0.6 (3)
O1—C13—C14—F6	62.7 (4)	O2—Co1—N2—C12	88.0 (2)
C15—C13—C14—F6	−116.0 (3)	O4—Co1—N2—C12	−90.1 (2)
O1—C13—C14—F5	−177.3 (3)	O1—Co1—N2—C12	−0.2 (2)
C15—C13—C14—F5	3.9 (4)	N1—Co1—N2—C12	178.7 (2)
O1—C13—C15—C16	−2.0 (5)	O2—Co1—N2—C8	−91.62 (19)
C14—C13—C15—C16	176.6 (3)	O4—Co1—N2—C8	90.35 (19)
C13—C15—C16—O2	5.1 (5)	O1—Co1—N2—C8	−179.74 (19)
C13—C15—C16—C17	−170.3 (3)	N1—Co1—N2—C8	−0.91 (18)
O2—C16—C17—F1	166.4 (3)	C15—C13—O1—Co1	−16.6 (4)
C15—C16—C17—F1	−17.5 (5)	C14—C13—O1—Co1	164.79 (19)
O2—C16—C17—F3	37.7 (4)	O2—Co1—O1—C13	23.0 (2)
C15—C16—C17—F3	−146.2 (3)	O4—Co1—O1—C13	−151.3 (2)
O2—C16—C17—F2	−76.6 (4)	O3—Co1—O1—C13	−64.9 (2)
C15—C16—C17—F2	99.4 (3)	N2—Co1—O1—C13	117.9 (2)
O3—C18—C19—F7	−67.9 (5)	N1—Co1—O1—C13	111.4 (5)
C20—C18—C19—F7	112.6 (4)	C15—C16—O2—Co1	11.3 (4)
O3—C18—C19—F9	168.8 (3)	C17—C16—O2—Co1	−173.2 (2)
C20—C18—C19—F9	−10.7 (5)	O1—Co1—O2—C16	−20.5 (2)
O3—C18—C19—F8	51.9 (4)	O3—Co1—O2—C16	64.7 (2)
C20—C18—C19—F8	−127.5 (3)	N2—Co1—O2—C16	−113.2 (2)
O3—C18—C20—C21	−5.0 (5)	N1—Co1—O2—C16	169.6 (2)
C19—C18—C20—C21	174.4 (3)	C20—C18—O3—Co1	−16.7 (4)
C18—C20—C21—O4	2.5 (5)	C19—C18—O3—Co1	163.9 (2)
C18—C20—C21—C22	−174.7 (3)	O2—Co1—O3—C18	−150.4 (2)
O4—C21—C22—F11	−118.6 (4)	O4—Co1—O3—C18	27.8 (2)
C20—C21—C22—F11	59.0 (4)	O1—Co1—O3—C18	−62.3 (2)
O4—C21—C22—F12	3.9 (4)	N1—Co1—O3—C18	118.3 (2)
C20—C21—C22—F12	−178.4 (3)	C20—C21—O4—Co1	21.0 (4)
O4—C21—C22—F10	122.6 (3)	C22—C21—O4—Co1	−161.8 (2)
C20—C21—C22—F10	−59.7 (4)	O1—Co1—O4—C21	55.7 (2)
C8—C7—N1—C4	177.8 (2)	O3—Co1—O4—C21	−29.4 (2)
C8—C7—N1—Co1	−3.7 (3)	N2—Co1—O4—C21	148.6 (2)
C3—C4—N1—C7	−18.7 (4)	N1—Co1—O4—C21	−134.3 (2)
